# The role of CD36-Fabp4-PPARγ in skeletal muscle involves insulin resistance in intrauterine growth retardation mice with catch-up growth

**DOI:** 10.1186/s12902-021-00921-4

**Published:** 2022-01-04

**Authors:** Jing Liu, Hang Zhao, Linlin Yang, Xing Wang, Linquan Yang, Yuling Xing, Xiuqin Lv, Huijuan Ma, Guangyao Song

**Affiliations:** 1 Department of Internal Medicine, Hebei Medical University, Shijiazhuang, 050017 Hebei China; 2 Department of Endocrinology, Hebei General Hospital, Shijiazhuang, 050051 Hebei China; 3 Hebei Key Laboratory of Metabolic Diseases, Shijiazhuang, 050051 Hebei China; 4 Clinical Medicine Research Center, Hebei General Hospital, Shijiazhuang, 050051 Hebei China

**Keywords:** Low birth weight, CD36, Insulin resistance

## Abstract

**Background:**

Studies have shown that the high incidence of type 2 diabetes in China is associated with low birth weight and excessive nutrition in adulthood, which occurred during the famine years of the 1950s and 1960s, though the specific molecular mechanisms are unclear. In this study, we proposed a severe maternal caloric restriction during late pregnancy, followed by a post weaning high-fat diet in mice. After weaning, normal and high-fat diets were provided to mice to simulate the dietary pattern of modern society.

**Methods:**

The pregnant mice were divided into two groups: normal birth weight (NBW) group and low birth weight (LBW) group. After 3 weeks for weaning, the male offspring mice in the NBW and LBW groups were then randomly divided into four subgroups: NC, NH, LC and LC groups. The offspring mice in the NC, NH, LC and LC groups were respectively fed with normal diet, normal diet, high-fat diet and high-fat diet for 18 weeks. After 18 weeks of dietary intervention, detailed analyses of mRNA and protein expression patterns, signaling pathway activities, and promoter methylation states were conducted for all relevant genes.

**Results:**

After dietary intervention for 18 weeks, the expressions of CD36, Fabp4, PPARγ, FAS, and ACC1 in the skeletal muscle tissue of the LH group were significantly increased compared with the LC and NH groups (*P* < 0.05). The level of p-AMPK/AMPK in the skeletal muscle tissue of the LH group was significantly decreased compared with the LC and NH groups (*P* < 0.05). CPT1 and PGC-1α protein expressions were up-regulated in the LH group (*P* < 0.05) compared to the LC group. Additionally, the DNA methylation levels of the PGC-1α and GLUT4 gene promoters in the skeletal muscle of the LH groups were higher than those of the LC and NH groups (*P* < 0.05). However, PPARγ DNA methylation level in the LH group was lower than those of the LC and NH groups (*P* < 0.05).

**Conclusions:**

LBW combined with high-fat diets may increase insulin resistance and diabetes through regulating the CD36-related Fabp4-PPARγ and AMPK/ACC signaling pathways.

## Background

Low birth weight (LBW) is an important indicator of fetal environmental damage during intrauterine growth. According to the statistics of the World Health Organization, more than 20 million newborns weigh less than 2500 g, where the overall incidence of LBW in China is 2.18%, with an increasing trend year by year [1]. LBW not only increases the risk of morbidity and mortality in the perinatal period, but also increases the risk of metabolic diseases in adulthood, such as insulin resistance (IR), type 2 diabetes, and obesity [2–4]. A previous meta-analysis study used 21 previous clinical studies (313,165 clinical samples), and found that LBW individuals have a significantly increased risk of developing type 2 diabetes and IR compared with other birth weights, as well as IR might be the central pathogenesis of these chronic metabolic diseases [5]. Ye et al. observed that catch-up growth in early childhood and disorders involving in glucose and lipid metabolism in adulthood were accompanied by impaired IGFBP3/IGF-1/IRS-1/Akt signaling pathway in the liver [6]. In the early stage of our study, we found that deoxycholic acid and cholic acid levels in the blood of LBW mice were significantly lower than those of normal birth weight mice, suggesting that cholic acid metabolism might play a role in adult type 2 diabetes mellitus caused by LBW [7]. However, the mechanisms inducing IR and the development of diabetes in LBW individuals remain unclear.

Skeletal muscle is the main peripheral tissue of glucose and lipid metabolism stimulated by islets, and plays an important role in maintaining the balance of mechanical energy and quantity. IR in skeletal muscle is an important characteristic of type 2 diabetes. The imbalance between fatty acid uptake and oxidation is the main cause of lipid deposition and IR in skeletal muscle. Mitochondrial injury and fatty acid oxidation (FAO) disorders in skeletal muscle induced by a high-fat diet are important factors of IR. The AMPK/ACC signaling pathway plays an important role in regulation of fatty acid oxidation. CD36, known as the fatty acid translocation enzyme, is a type of single chain transmembrane glycoprotein, and widely expressed in various tissues and cells. As a carrier of fatty acids, CD36 protein expression level is associated with impaired fatty acid uptake, oxidation, and IR [8, 9]. Besides, CD36 signaling has been reported to regulate FAO by directly modulating AMPK activation [9].

Studies have shown that the CD36- and FABP4-mediated lipid transport pathways play important roles in the development of IR and atherosclerosis [3]. Long-chain fatty acid (LCFA) is an important ligand of PPARγ, which can be activated after transport to the nucleus by the Cd36-Fabp4 pathway, thus promoting glycolysis and lipid metabolism and increasing insulin sensitivity [10, 11]. The uptake of glucose in the skeletal muscles is mainly regulated by insulin-responsive glucose transporter 4 (GLUT4) and PPARγ coactivator-1α (PGC-1α). GLUT4 and PGC-1α can mediate the expression of ATP synthesis and oxidative phosphorylation genes.

The increased susceptibility to diabetes may be largely due to the generation of the *PGC-1* transcriptional coactivator, accompanied by the phenotypic changes (primarily through epigenetic changes) observed in rat offspring induced by environmental factors in the embryonic period. The changes of the gene sequences are not responsible for the epigenetic regulation of gene expression, and instead, the modifications of gene structure through DNA methylation, chromatin remodeling, and histone modifications can regulate epigenetics.

The mechanisms of CD36 regulating muscle glucose metabolism and IR in LBW mice are unknown. This study aimed to investigate the dynamic changes in insulin sensitivity, the CD36-related Fabp4-PPARγ and AMPK/ACC signaling pathways in skeletal muscle tissues, and the relationship between catch-up growth and IR in SGA mice.

## Methods

### Animals and experimental design

The animal experiment was conducted in compliance with the relevant guidelines and regulations and was approved by the Animal Ethics Committee of Hebei General Hospital. Adult ICR mice aged 6–8 weeks were selected for this experiment. After one week of free access to water and adaptive feeding, the female and male mice were raised in the same cage at a ratio of 2:1. On the second day, the female mice were observed to determine whether they were pregnant through the presence or absence of vaginal plug, and the appearance of vaginal plug was recorded as 0.5 day of gestatin. On the 12.5 day of pregnancy, ICR pregnant mice were randomly divided into normal birth weight (NBW) group and LBW group.

From the 12.5 day to 18.5 days of pregnancy, the mice in the NBW group was fed with a normal diet (free diet), while the mice in the LBW group was provided with a 50% diet restriction (intake calculated according to the NBW group). After birth, the number of newborn mice was recorded along with their weights. In order to ensure the balance of breast feeding, the litter number less than 8 were all discarded, and the litter number more than 8 retained 8 according to the principle of removing the highest and the lowest weight.

**Fig. 1:**
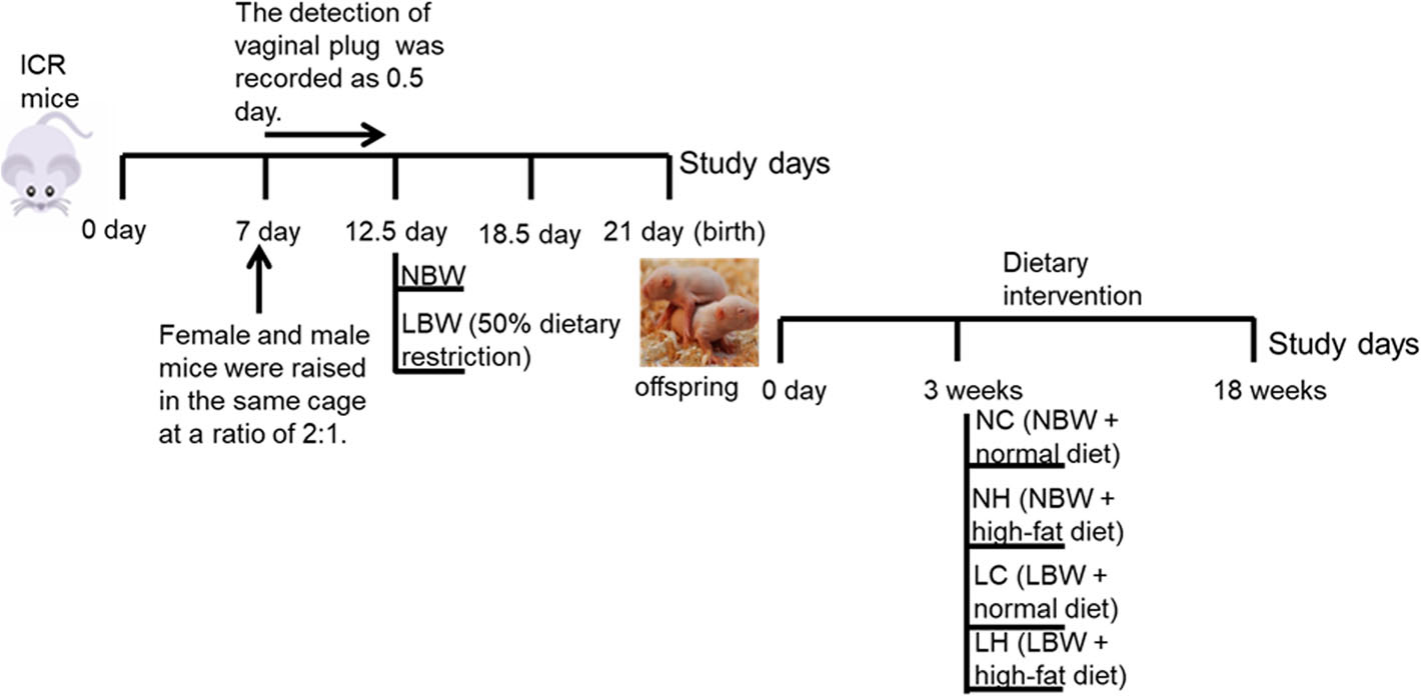
The timeline of animal experiments

After 3 weeks, the selected weanlings in the NBW and LBW groups were randomly divided into the following four subgroups (*n* = 9): the normal birth weight + normal diet group (NC), normal birth weight + high-fat diet group (NH), low birth weight + normal diet group (LC), and low birth weight + high-fat diet group (LH). The mice in the NC, NH, LC and LH groups were respectively fed with normal diet, normal diet, high-fat diet and high-fat diet for 18 weeks. The normal diet D12450J and the high-fat diet D12492 were prepared and purchased from Beijing Huafukang Biotechnology Co., Ltd. (Beijing, China). Among them, normal diet contains 20% protein, 70% carbohydrate,10% fat, and 3.85 kcal/g calorific value; high-fat diet includes 20% protein, 20% carbohydrate, 60% fat, and 5.24 kcal/g calorific value. The timeline of all animal experiments are shown in Fig. 1.

### Measurement of glucose metabolic and IR indices

During 18 weeks of dietary intervention, the body weight and food intake of the mice were recorded every week. Meanwhile, the intra-abdominal glucose tolerance test (IPGTT) was performed at the end of 8, 12, and 16 weeks following intervention. The blood glucose level was measured at 0, 15, 30, 60, and 120 min; and the area under glucose curve (AUC) was obtained to evaluate the degree of IR. Finally, the mice were sacrificed, and the skeletal muscle tissue was isolated from the mice and preserved at − 80 °C. Serum samples were collected, and then fasting blood glucose (FBG) levels and fasting insulin (FINS) levels were determined using a blood glucose determination kit and immunoradiometric assay, respectively. After that, a Homoeostasis Model Assessment for Insulin Resistance (HOMA-IR) was calculated according to the following formula: HOMA-IR = FINS (mU/L) × FBG (mmol/L)/ 22.5, where the value was adjusted further through a logarithmic transformation.

### Reverse transcription-quantitative polymerase chain reaction (RT-qPCR)

Total RNA was extracted from skeletal muscle tissue using the RNAsimple Total RNA Kit (TIANGEN DP419, China) and the mRNA expression levels of CD36, Acetyl-CoA carboxylase 1(*ACC1*), fatty acid synthase (*FAS*), peroxisome proliferator-activated receptor γ (*PPARγ*), and peroxisome proliferator activated receptor γ coactivator-1α (*PGC-1α*) were measured using 2^−ΔΔCt^ method. The all primers were purchased from Integrated DNA Technology (Table 1). The TaqMan assay reagents and endogenous control were purchased from Applied Biosystems and were used to measure Ct values using the ABI Prism 7500 PCR system (Applied Biosystems, Shijiazhuang, China). Normalizations of the target gene expression were performed using *GAPDH* as a reference gene.

**Table 1:** Primer sequences

Primer	Sequence (5′ → 3′)
**PPARγ**	F: CTGACCCAATGGTTGCTGATTAC R: GGACGCAGGCTCTACTTTGATC
**Fabp4**	F: CCCTGCCATTGTTAAGACC R: TGCTGCTGTTCCTGTTTTC
**CD36**	F: GGCTAAATGAGACTGGGACC R: CAAACATCACCACTCCAATCC
**FAS**	F: ATCTGGGCTGTCCTGCCTCT R: CAGTTTCACGAACCCGCCT
**ACC1**	F: GCTAAAGGGCGATCTCAACAAAG R: TTCTCTCCGTGGTTAGGGTTCT
**PGC-1a**	F: CAAGACCAGGAAATCCGAG R: TGAAGTCGCCATCCCTTAG

### Western blot assay

Total proteins were extracted from the skeletal muscle tissues using the Automatic sample freezing grinder (Shanghai Jing Xin, China, JXFSTPRP-CL). A total of 220 to 250 mg of skeletal muscle tissue was extracted from each sample. 1 mL protein lysate precooled to 4 °C was added. The homogenates were centrifuged at 13,000 rpm for 5 min (3 times) at 4 °C according to the manufacturer’s instructions. The protein concentrations were evaluated using a BCA Protein Quantification Kit (Lot 23,227, ThermoFisher Technology co LTD, USA) following the manufacturer’s instructions. After that, 30 μg of protein for each sample was resolved and loaded on a 10% sodium dodecyl sulfate-polyacrylamide gel, electrophoresed, and subsequently electroblotted onto polyvinylidene difluoride (PVDF) membranes. The membrane was then blocked with 5% skim milk for 2–4 h at room temperature and subsequently incubated with primary antibody anti-CD36 1:1000 (Abcam, ab133625), anti-AMPK 1:1000 (arigo Biolaboratories Corp, ARG51172), anti-FAS 1:1000 (Cell Signaling Technology, Inc., 3180), anti-ACC1 1:1000 (Cell Signaling Technology, Inc., 3190), anti-PPARγ 1:1000 (Proteintech Group, Inc., 16,643–1-AP), anti-CPT1 1:1000 (Aviva, ARP44796_P050), anti-FABP4 1:1000 (Cell Signaling Technology, Inc., 3544), anti-PGC-1α 1:1000 (Cell Signaling Technology, Inc., 2178), and GAPDH 1:5000 (bioworld, AP003) overnight at 4 °C, respectively.

The prepared antibody diluent was used to dilute HRP-labeled goat anti-rabbit IgG according to the secondary antibody dilution ratio (1:500–1:10,000). After overnight incubation with the primary antibody, the PVDF membrane was immersed in TBST solution and placed on a shaking table for rinsing (3 rinses, 10 min each). The membrane was then placed in a plastic hybridization bag, treated with the corresponding diluted secondary antibody, and incubated by placing on the shaking table at 37 °C for 1 h. Protein bands were visualized with a gel imager, and the protein expressions were quantified using the ImageJ software.

### DNA methylation

Genomic DNA from rat skeletal muscle was extracted using the EZ DNA Methylation-Gold™ Kit (Beijing Tianmo) according to the manufacturer’s instructions. The sequences of all primers were designed using the PyroMark primer design software and synthesized by Sangon Biological Co. Ltd. (Shanghai, China). The sequences of *PPARγ*, *PGC-1α*, and *GLUT4* are shown in Tables 2, 3 and 4.

**Table 2:** Primer design for the PGC-1*α* assay

PGC-1*α* primo* assay, designed by PyroMark, Qiagoi	
**Amplicon product**	
**Forward primer**	TGTAGGAGATTTGAGTTATTATGTGAG
**Reverse primer**	ACCTTTAAAAAACTTCAAACATCAC
**Sequencing primer**	GAGTTATTATGTGAGTAGGGTTT
**Sequence intended for analysis**	TGTAGGAGATTTGAGTTATTATGTGAGTAGGGTTTCGGTTTAGAGTTGGTGGTATTTAAAGTTGGTTTTAGTTATAGTGTGATGTTTGAAGTTTTTTAAAGGT (103 bp)

**Table 3:** Primer design for the PPARγ assay

PPARγ primo* assay, designed by PyroMark, Qiagoi	
**Amplicon product**	
**Forward primer**	TGTGTGATTAGGAGTTTTAATTAAAG
**Reverse primer**	ACCTTAATCTCTAAATTATAAAACACC
**Sequencing primer**	TCTAAATTATAAAACACCAAATAAA
**Sequence intended for analysis**	GTTTGGGATAGGTTGGGATATTCGGGATTTGATATTTGGCGGAGTTTAACGTGGGAATTAAAAATAGTTATTTCGGGTTATTTCGGGGTATATATATATATATATATATATATATATATATATACGCGGGTTTTATGTTATTTTGTTGGAGTTATT (93 bp)

**Table 4:** Primer design for the Glut4 assay

Glut4 primo* assay, designed by PyroMark, Qiagoi	
**Amplicon product**	
**Forward primer**	GTTTGGGATAGGTTGGGATAT
**Reverse primer**	AATAACTCCAACAAAATAACATAAAAC
**Sequencing primer**	ATATTAGGGATTTGATATTTGG
**Sequence intended for analysis**	GTTTGGGATAGGTTGGGATATTCGGGATTTGATATTTGGCGGAGTTTAACGTGGGAATTAAAAATAGTTATTTCGGGTTATTTCGGGGTATATATATATATATATATATATATATATATATATACGCGGGTTTTATGTTATTTTGTTGGAGTTATT (156 bp)

After that, PyroMark PCR kit (Qiagen) including 50 ng DNA (muscle), PCR primers, Taq enzyme, dNTPs, and Taq Buffer was used for PCR based on the manufacturer’s protocols. The PCR conditions were displayed as follows: 95 °C for 5 min, a total of 50 cycles at 94 °C for 30 s, followed by 55 °C for 30 s, then 72 °C for 30 s, and with a final extension at 72 °C for 8 min. Finally, the PCR product (5 μl) was resolved by electrophoresis on a 1% agarose gel to confirm identity of the product, and then sent to Sangon Biological Co. Ltd. for sequencing using the PyroMark Q96 ID pyrosequencing machine (Qiagen).

For pyrosequencing, the PPARγ primer and PGC-1α both target 1 CpG site, as well as the GLUT4 primer targets 4 CpG sites. The PCR products were sequenced, and DNA methylation rations were calculated through determining relative peck heights of cytosine (C) and thymine (T) peaks at each CpG site. The methylation ratios were calculated as C/(C + T), and the data were expressed as percent methylation [12–14].

### Statistical analysis

Results are presented as mean ± SD. Differences within groups were analyzed using an ANOVA, followed by the Kruskal-Wallis nonparametric test analysis. The value of *P* < 0.05 was considered statistically significant. All statistical analyses were conducted with the SPSS statistical software, version 21.0.

## Results

### Establishment of a LBW model

**Fig. 2:**
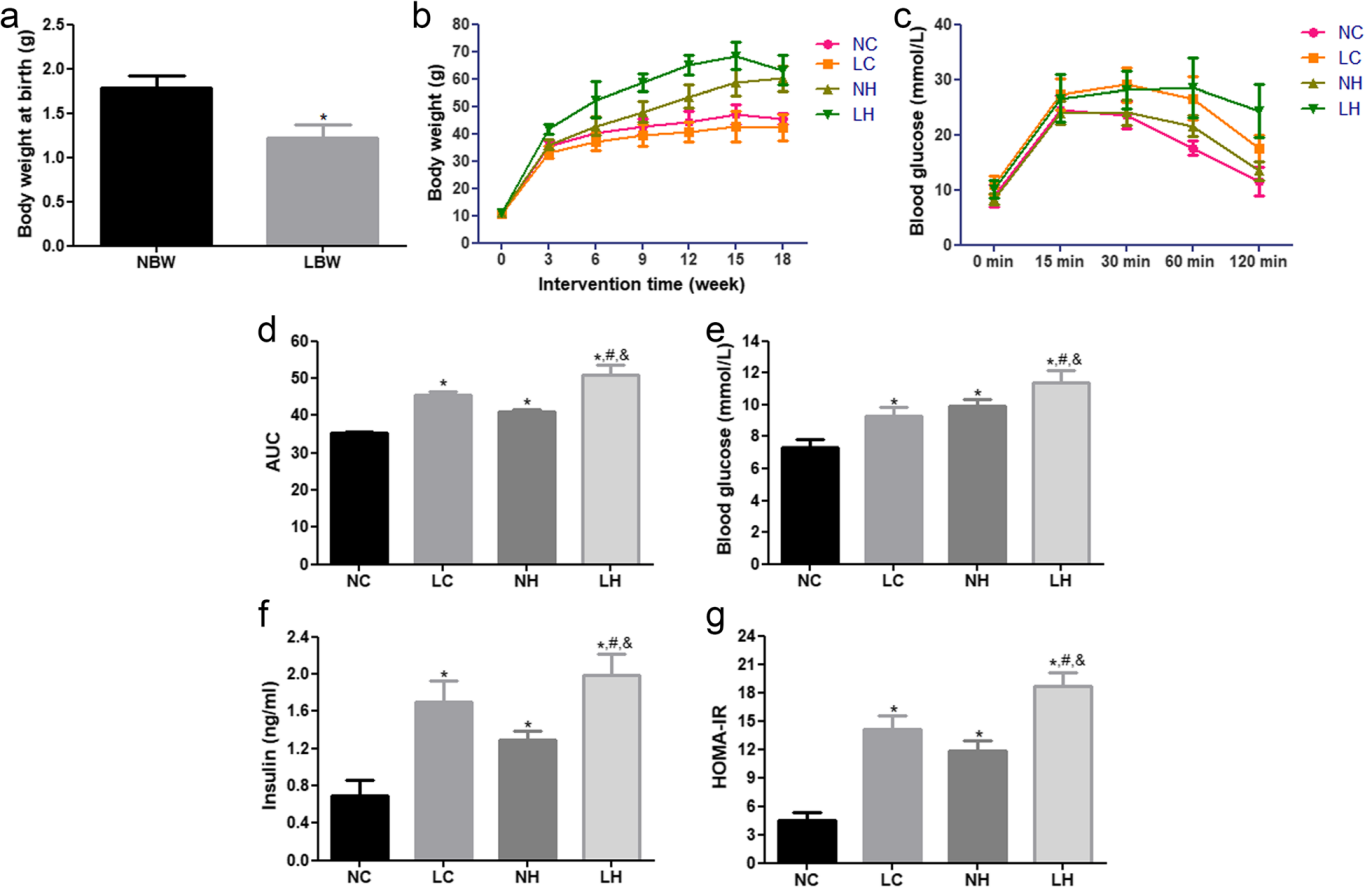
The body weight, blood glucose, fast insulin, and HOMA-IR in each group at the end of 18 weeks of dietary intervention. **A** The birth weight of offspring mice was significantly lower than that of pregnant mice without dietary restriction. **B** The body weight growth of the mice in different groups. C The blood glucose level at each time point of the IPGTT test during the 16 weeks of dietary intervention. **D** The area under glucose curve (AUC) during the 16 weeks of diet intervention. **E** The blood glucose in mice of each group.F The insulin in mice of each group. **G** HOMA-IR in mice of each group. NC: normal birth weight fed a normal diet; LC: low-birth-weight mice fed with a normal diet; NH: normal birth weight mice fed with a high-fat diet; LH: low birth weight mice fed with a high-fat diet. *P < 0.05 vs NC group, ^#^*P* < 0.05 vs LC group, ^&^*P* < 0.05 vs NH group

In this study, 50% dietary restriction was ensured for pregnant mice in the middle and late stages of pregnancy (12.5 to 18.5 days). It is clear that the birth weight of offspring mice in the pregnant mice with dietary restriction (1.30 ± 0.07 g) was significantly lower than that of pregnant mice with normal diet (*P* < 0.05), indicating that the LBW model was successfully established (Fig. 2A).

### LBW offspring develop IR and disrupted glucose regulation

At the end of the experiment, the final body weight of the mice in the NH and LH groups was significantly higher than that in the NC group (*P* < 0.05), and the body weight growth of the four groups is shown in Fig. 2B. Furthermore, there was no statistically significant difference in the weekly dietary intake among the NC, LC, NH and LH groups (data not shown).

After weaning, the selected male mice in the NC, NH, LC and LH groups were fed with different diets for 18 weeks. At the end of 16 weeks, the blood glucose level in the LH group was significantly higher (at 0, 30, and 120 min) than that in the NH group (*P* < 0.05), and the area under the glucose curve (AUC) was consistent with the trend of increasing blood glucose (*P* < 0.05, Fig. 2C, D). At the end of 18 weeks, the serum levels of FBG and FINS in the NH and LH groups were higher than those in the NC group (*P* < 0.05, Fig. 2E, F). Compared with the NH group, the FBG and FINS levels in the LH group were also evidently higher (*P* < 0.05). Thereafter, the values of HOMA-IR were calculated. The results showed that the values of HOMA-IR in the NH and LH groups were significantly higher than that in the NC group (*P* < 0.05), and the value of HOMA-IR in the LH group was markedly higher than that in the NH group (*P* < 0.05, Fig. 2G). All these results indicated that LBW mice fed with high-fat diet exhibited severely disrupted regulation of glucose and IR in adulthood.

### Changes in the expression of CD36-Fabp4-PPARγ pathway-related proteins and genes in skeletal muscle of the mice in each group

**Fig. 3:**
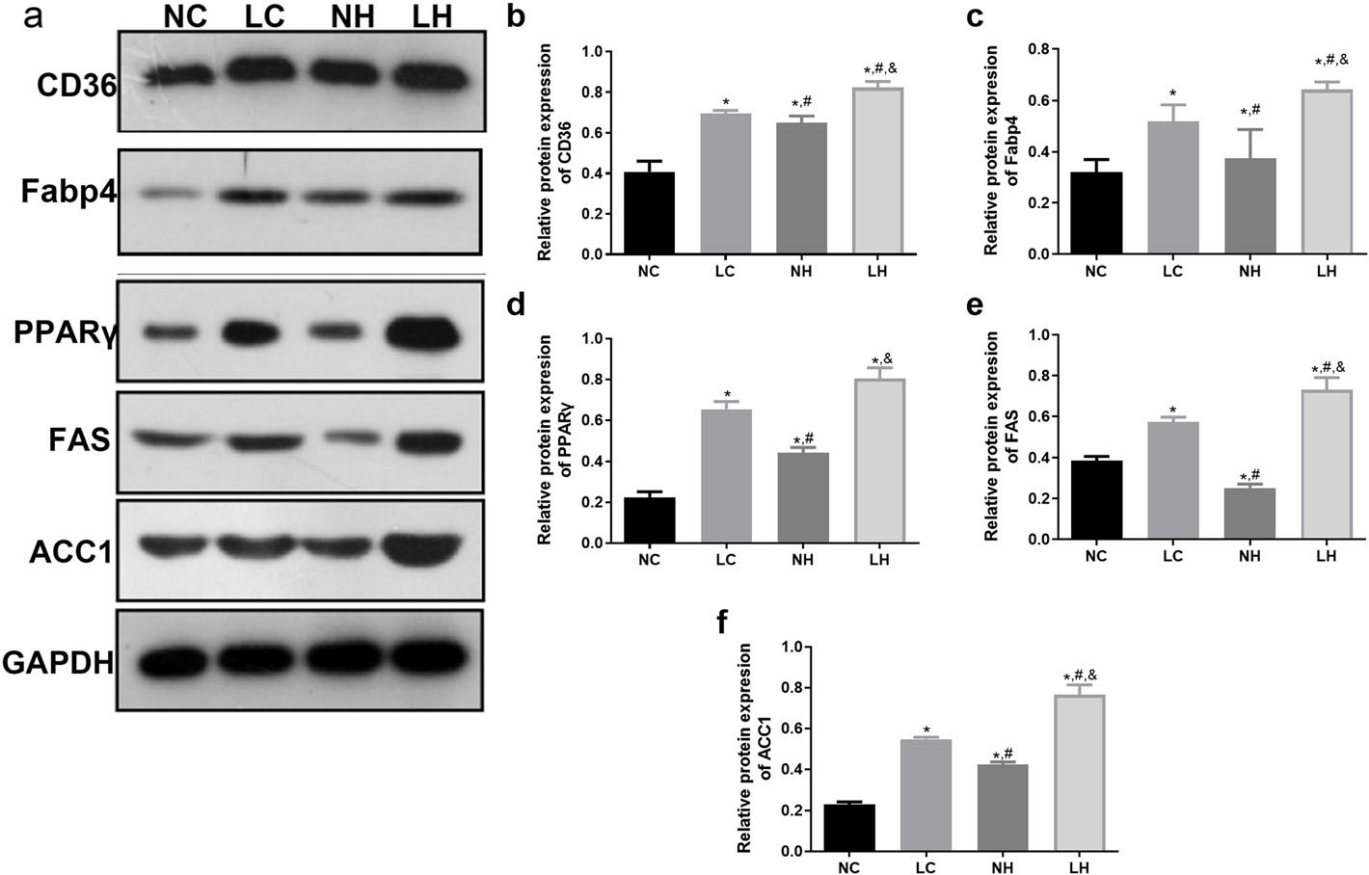
The protein expressions of CD36, Fabp4, PPARγ, ACC1, and FAS in the NC, LC, NH, and LH groups determined by western blot. **A** The protein bands visualized by western blot. **B** The relative protein expression of CD36. **C** The relative protein expression of Fabp4. **D** The relative protein expression of PPARγ. **E** The relative protein expression of FAS. **F** The relative protein expression of ACC1.Data are mean ± SD(*n* = 3)*P < 0.05 vs NC group, ^#^*P* < 0.05 vs LC group, ^&^*P* < 0.05 vs NH group

**Fig. 4:**
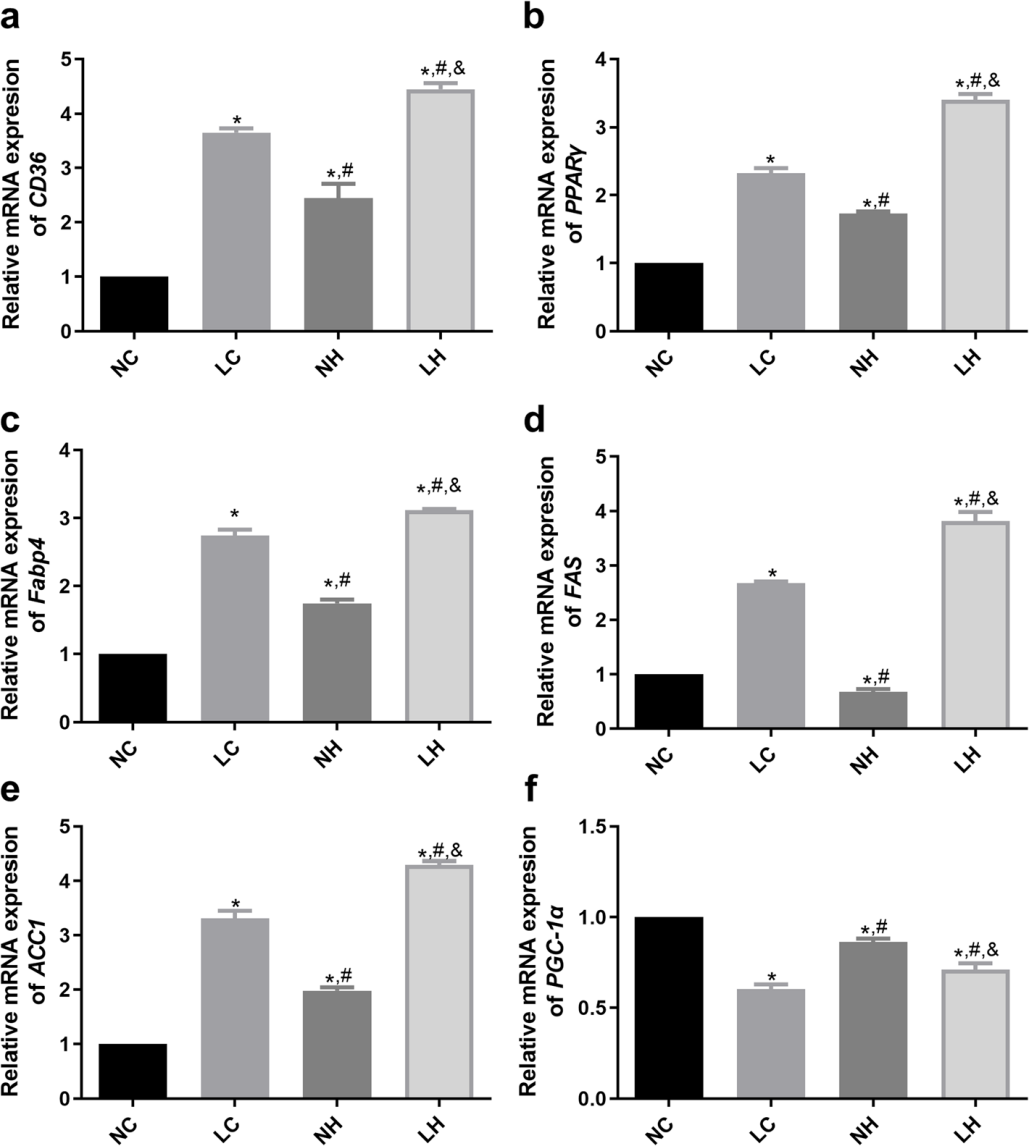
The mRNA expressions of *CD36*, *PPARγ*, *Fabp4*, *ACC1*, *FAS* and *PGC-1α* in the NC, LC, NH, and LH groups measured by reverse transcription-quantitative polymerase chain reaction. **A** The mRNA expression of *CD36*. **B** The mRNA expression of *PPARγ*. **C** The mRNA expression of *Fabp4*. **D** The mRNA expression of *FAS*. **E** The mRNA expression of *ACC1*. **F** The mRNA expression of *PGC-1α*.Data are mean ± SD(*n* = 3)*P < 0.05 vs NC group, ^#^*P* < 0.05 vs LC group, ^&^*P* < 0.05 vs NH group

After 18 weeks of high-fat diet intervention, the protein and mRNA expression levels of CD36, Fabp4, PPARγ, ACC1, and FAS in the skeletal muscle tissue of mice were determined. It is clear that the protein expression levels of CD36, Fabp4, PPARγ, ACC1 and FAS in the LC group was significantly higher than those in the NC group (*P* < 0.05, Fig. 3A-F). Compared with the NC group, the protein expressions of CD36, PPARγ, Fabp4, and ACC1 in the NH group were significantly up-regulated (*P* < 0.05); while the protein expressions FAS was evidently down-regulated (*P* < 0.05, Fig. 3A-F). However, their protein expressions in the LH group were all significantly up-regulated compared with the LC and NH groups (*P* < 0.05, Fig. 3A-F). Additionally, the trends of CD36, Fabp4, PPARγ and ACC1 mRNA expressions in different groups measured by RT-qPCR were similar with those detected by western blot (Fig. 4A-E). Furthermore, compared with the NC group, the mRNA expression of *PGC-1α* was significantly down-regulated in the LC, NH and LH groups (*P* < 0.05); while its mRNA expression in the NH group was evidently higher than that in the LC and LH groups (P < 0.05, Fig. 4F).

### Changes of expression of AMPK/ACC signaling pathway-related proteins in skeletal muscle of the mice in each group

**Fig. 5:**
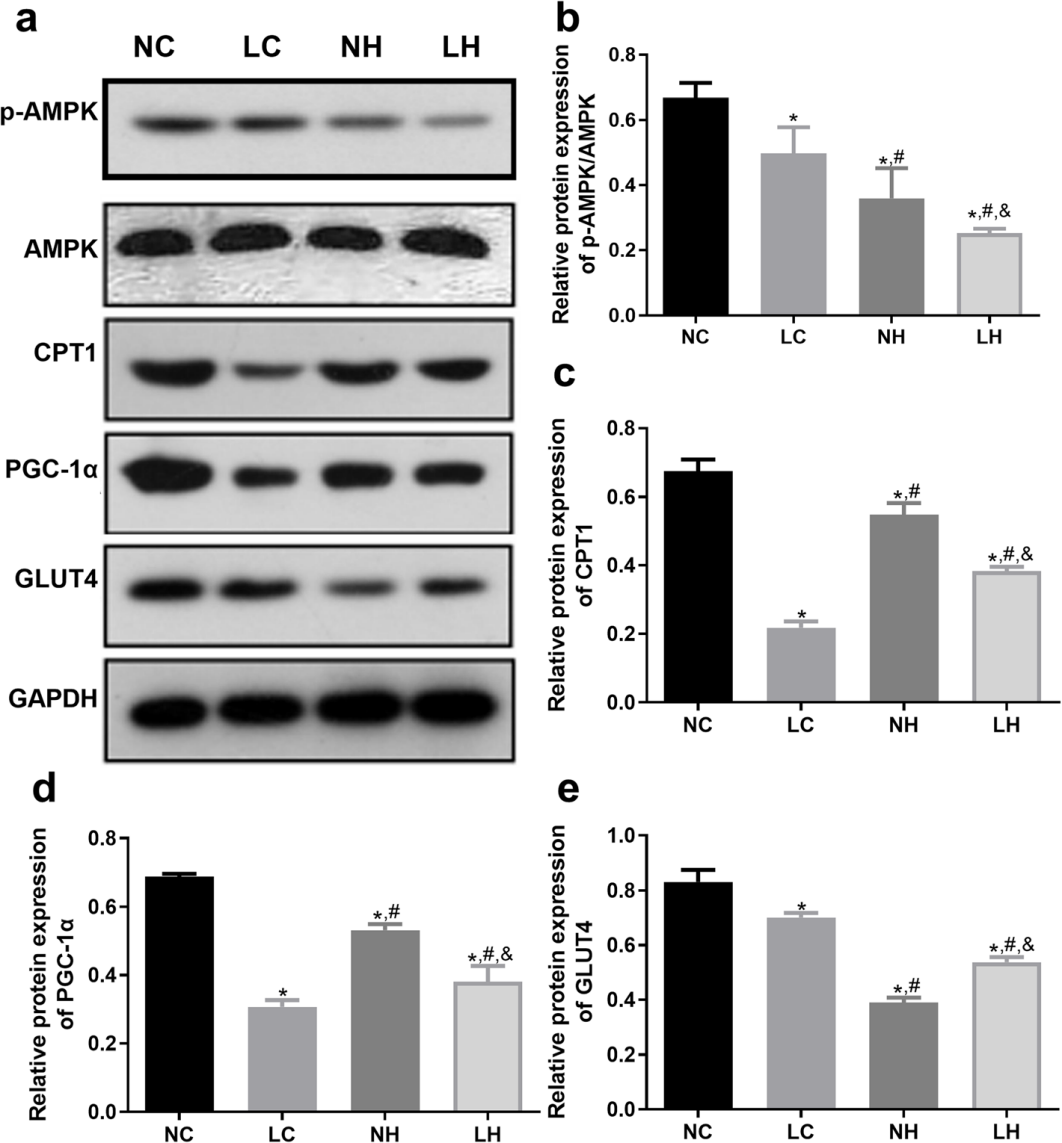
The protein expressions of p-AMPK, AMPK, PGC-1α, CPT1, and GLUT4 in the NC, LC, NH, and LH groups determined by western blot. **A** The protein bands visualized by western blot. **B** The level of p-AMPK/AMPK. **C** The relative protein expression of CPT1. **D** The relative protein expression of PGC-1α. **E** The relative protein expression of GLUT4.Data are mean ± SD(*n* = 3)*P < 0.05 vs NC group, ^#^*P* < 0.05 vs LC group, ^&^*P* < 0.05 vs NH group

After that, the protein expressions of p-AMPK, AMPK, CPT1, PGC-1α and GLUT4 were further analyzed by western blot. It is clear that the level of p-AMPK/AMPK was lower in the LC group than that in the NC group (*P* < 0.05), its level in the LH group was decreased compared with the NH and LC groups (*P* < 0.05, Fig. 5A, B). For CPT1 and PGC-1α, their expressions were significantly down-regulated in the LC, NH and LH groups compared to the NC group (*P* < 0.05); and their expressions were higher in the NH and LH groups than those in the LC group (*P* < 0.05, Fig. 5A, C, D). Compared with the NH group, CPT1 and PGC-1α expressions were down-regulated in the LH group (*P* < 0.05, Fig. 5A, C, D). The tendency of GLUT4 protein expression in different groups was similar to that of p-AMPK/AMPK level.

### DNA methylation

**Fig. 6:**
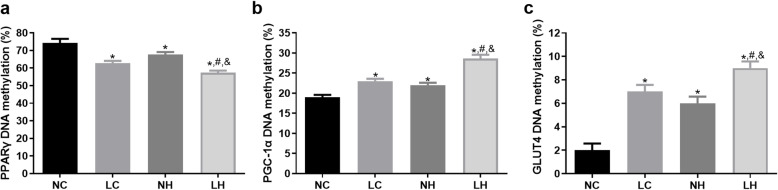
The DNA methylation ratio of PPARγ, PGC-1α, and GLUT4 in the NC, LC, NH, and LH groups. **A** The DNA methylation ration of PPARγ. **B** The DNA methylation ration of PGC-1α. **C** The DNA methylation ration of GLUT4. *P < 0.05 vs NC group, ^#^*P* < 0.05 vs LC group, ^&^*P* < 0.05 vs NH group

The DNA methylation levels of PPARγ, PGC-1α and GLUT4 were further detected. The PPARγ DNA methylation level was increased in the LH group compared with the LC and NH groups (*P* < 0.05, Fig. 6A). For PGC-1α, its DNA methylation rates in the NC, LC,NH and LH groups were 19 ± 1%, 23 ± 1%, 20 ± 1%, and 28.67 ± 1.53%, respectively (Fig. 6B). Compared with the NC group, the PGC-1α DNA methylation level in the LC, NH and LH groups were significantly higher (*P* < 0.05), and the level in the LH group was more significant (*P* < 0.05). Additionally, the tendency of GLUT4 DNA methylation level in different groups was similar to that of PGC-1α DNA methylation level (Fig. 6C).

## Discussion

In our study, the LBW model was constructed by restricting the diet of female mice in the middle and third trimester of pregnancy, similar to the methods used by other investigators [2]. The birth weights of the offspring from mice with dietary restriction were significantly lower than those from mice with normal diet, which demonstrated successful establishment of the LBW mice model. Barker et al. [15] firstly proposed the “thrifty phenotype” hypothesis, stating that the fetal under-nutrition can compel the body to store fat (after improvement of the nutritional environment) rather than muscle. This adaptive adjustment in the fetus is more likely to lead to obesity, IR, and type 2 diabetes [7]. Our study found that in the LBW mice with a high-fat diet, glucose regulation was more seriously disrupted in adulthood and IR was more evident, which were consistent with the findings of previous studies [6, 16].

Mitochondrial damage and abnormal fatty acid metabolism (especially) usually contribute to IR [17, 18]. The skeletal muscle represents the main target tissue of insulin, and is the most important peripheral tissue stimulated by insulin [19]. CD36 is widely expressed in a variety of tissues and cells, and functions as a fatty acid translocation enzyme that is a single chain transmembrane glycoprotein. As a fatty acid transport carrier, the expression of CD36 in skeletal muscle membrane is an important indicator of fatty acid uptake and oxidation activity. CD36 also modulates the role of insulin to stimulate muscle glucose utilization and to influence postprandial glucose metabolism [20]. Samovski et al. indicated that high-fat diet can induce an increase of CD36 protein level in the cell membrane of skeletal muscle cells, and CD36-deficient mice show an increased peripheral tissue sensitivity to insulin, due to reduced skeletal muscle fatty acid uptake [10]. In our study, the level of CD36 in the skeletal muscle tissue of LBW mice with the high-fat diet was significantly increased compared with the LBW mice with the normal diet. Moreover, we found that the CD36 protein level in the skeletal muscle tissue of LBW mice with the high-fat diet was higher than that in the normal weight mice with the high-fat diet. A previous study confirmed the association between early life exposure to malnutrition and the development of long-term metabolic diseases through multiple epidemiological experiments, and proposed the “fetal programming hypothesis” that early intrauterine nutrition can influence fetal genome expression and may persist throughout life [21]. In addition, dietary habits after birth also have important effects on the occurrence and development of metabolic diseases in adulthood. Studies have shown that LBW offspring given a high-fat diet after birth were more prone to hyperinsulinemia and insulin resistance, as well as increased blood glucose levels and lipid deposition in the liver [22, 23], which indicated that disorders of glucose and lipid metabolism in adulthood may result from a combination of intrauterine and postnatal environments. Combined with our results, it can be inferred that high level of CD36 expression in muscle may be associated with the incidence of type 2 diabetes, and IR of the LBW infants with high-fat diets.

Excess fat accumulation may result in the release of free fatty acid (FFA), which can induce IR at high concentrations. CD36 activation with hexarelin can promote mitochondrial activity and biogenesis through enhancing PPARγ and co-activator PGC-1α transcriptional activity [24]. CD36 has a high affinity for LCFA, and facilitates its entry into the cells, while the binding of LCFA to Fabp4 improves its water solubility [5]. PPARγ, an important transcription factor, regulates many transcriptional pathways related to adipogenesis, and can be activated after transport to the nucleus via the CD36-Fabp4 pathway. Besides, PPARγ has been reported to play important roles in fetal growth and development, as well as lipid and glucose metabolism. The lack of PPARγ activity is associated with a variety of diseases, such as diabetes, obesity, and high blood pressure. A research of Kelstrup et al. [25] has shown that the decreased gene expression of peroxisome proliferator-activated receptor gamma coactivator 1 alpha (PPARGC1A, also PGC-1α) in muscle was related to abnormal insulin function. High-fat overfeeding can increase PPARGC1A DNA methylation in a birth weight-dependent manner in muscle, while this methylation is flexible with different responses of muscle and fat [26]. Our study found that the methylation level of PGC-1α in the muscle of LBW mice was increased, and was further influenced by high-fat diet. Additionally, PGC-1α is also a coactivator of various transcription factors expressed in skeletal muscle, including PPARγ [27]. Lian et al. have reported that SGA individuals may exhibit high PPARγ expression, which may lead to abnormal fatty acidosis and lipid metabolism disorders [28].

In the present study, we also found that the protein and mRNA expression levels of Fabp4 and PPARγ in the skeletal muscle of LBW mice with high-fat diet were ehanced, compared with the normal mice with high-fat diet. After overfeeding with high-fat diets, the reduced methylation of PPARγ was found in the LH subject compared to the LC and NH groups. The CD36-Fabp4 pathway can activate PPARγ, thereby promoting glycolysis and lipid metabolism. ACC1, FAS, and PPARγ have been identified as major regulators of fatty acid biosynthesis. One of the important precursors in the fatty acids biosynthesis is the product of acetyl-CoA carboxylase (ACC), which is known as a mitochondrial fatty acid oxidation inhibitor, thereby playing a vital role in controlling lipid metabolism. FAS is a key enzyme in the synthesis of FFAs, and its abnormal expression is closely associated with the occurrence and development of obesity and IR [29]. Compared with the NH group, the ACC1 and FAS protein expression were up-regulated in the skeletal muscle of the LH group. These suggested that LBW can induce IR and abnormal glucose, while lipid metabolism may be regulated by CD36-Fabp4-PPARγ, thus affecting the FAO of skeletal muscle.

AMPK activation can stimulate the oxidation of fatty acids in skeletal muscle [30]. AMPK can reduce the synthesis of malonyl-CoA, fatty acids, and other lipids via inhibiting ACC phosphorylation. The fatty acid synthesis can be increased by reducing the inhibitory effect of malonyl-CoA on CPT-1α oxidative metabolism [31]. CPT1 is a key regulatory enzyme in skeletal muscle mitochondrial FAO, which can catalyze the formation of carnitine to form long-chain acyl carnitine and to activate fatty acid transfer in the mitochondria for oxidation [32, 33]. ACC1, as an upstream factor of CPT1, participates in the FAO process. The PGC-1α protein plays a major role in regulation of muscle oxidation, which affects the electron delivery system and uncouples the expression of related proteases. Expression of the nuclear respiration factors (NRFs) can regulate mitochondrial respiration and biosynthesis [34–37], which act as key factors in regulation of mitochondrial synthesis [38, 39]. GLUT4 is the entry point for glucose into muscles, and a rate-limiting step in glucose uptake, as well as its down-regulation may have essential effects on the development of decreased glucose tolerance in rats [40]. Interference with PGC-1α and GLUT4 expression in skeletal muscles has been reported to be closely related to IR in offspring [41]. In addition, the methylation CpG dinucleotides clusters in promoter regions of some genes may contribute to metabolic reprogramming. Zeng et al. [41] demonstrated that the CpG island methylation was significant in PGC-1α promoter sequence, as well as the down-regulation of PGC-1α might lead to the epigenetic modulation of PGC-1α in 18-month-old female offspring. Another study has shown that malnutrition continued to silence GLUT4 in utero possinly through the action of DNA methylation during aging, thus resulting in the age-related amplification of glucose intolerance.

Previous studies have found that a long-term high-fat diet could induce obesity and IR in rat skeletal muscle, consistent with the results from our study, while the level of p-AMPK/AMPK was significantly reduced in mice with high-fat diets [42, 43]. After the high-fat dietary intervention using, the CPT1, PGC-1α, p-AMPK/AMPK, and GLUT4 levels in the skeletal muscle of the NH and LH groups were significantly lower than those observed in the NC group, which was consistent with the previous findings [44, 45]. Furthermore, the levels of GLUT4 and PGC-1α mRNA and protein were decreased, accompanied by the increased methylation levels of GLUT4 and PGC-1α, compared with the NC groups. These also indicated that high-fat diet could cause not only heterotopic lipid deposition, but also result in the damage of skeletal muscle mitochondrial function. AMPK can enhance FA uptake and oxidation by promoting localization of the CD36 transporter to the cell membrane [9]. In our study, after the high-fat dietary intervention, CPT1 and PGC-1α protein expression was significantly reduced in mice, and the level of p-AMPK/AMPK was also decreased in the LH group, which suggested that LBW induced IR might be regulated by AMPK/CPT1 and AMPK/PGC-1α, thereby affecting the skeletal muscle lipid metabolism and mitochondrial function, and then inhibiting the mitochondrial oxidation of the FAO, eventually leading to the abnormal lipid accumulation.

## Conclusions

In conclusion, our study found that the abnormal glucose tolerance and IR are more evident in mice offspring with LBW after high-fat dietary consumption. Additionally, the CD36-Fabp4-PPARγ and AMPK-ACC-CPT1 signaling pathways may be related to IR and type 2 diabetes caused by catch-up growth.

## Data Availability

Data are available from the corresponding author upon reasonable request. The datasets used and/or analysed during the current study are available from the corresponding author on reasonable request.
